# Use of p48 flow diverters with hydrophilic polymer coating under prasugrel single antiplatelet therapy for intracranial aneurysms arising from small-caliber vessels (≤ 2 mm): case series, complication, and occlusion rates

**DOI:** 10.3389/fneur.2025.1605808

**Published:** 2025-06-24

**Authors:** Ali Khanafer, Pablo Albiña Palmarola, Kamran Hajiyev, Philip von Gottberg, Andrei Filioglo, Michael Forsting, Hans Henkes

**Affiliations:** ^1^Neuroradiologische Klinik, Klinikum Stuttgart, Stuttgart, Germany; ^2^Department of Neurosurgery, Institute of Emergency Care, Chisinau, Moldova; ^3^Medizinische Fakultät, Universität Duisburg-Essen, Essen, Germany

**Keywords:** flow diversion, aneurysm, small-caliber vessels, single antiplatelet therapy, coating and surface treatment

## Abstract

**Purpose:**

Flow-diverter (FD) stents have become an established treatment for intracranial aneurysms in recent years, but their use for aneurysms in distal cerebral vessels with small caliber remains controversial. This study reports our single-center experience in using hydrophilic polymer-coated (HPC) p48 MW FDs with prasugrel single antiplatelet therapy (SAPT) to treat ruptured and unruptured aneurysms arising from small-caliber vessels (≤ 2 mm).

**Methods:**

A prospectively maintained database was retrospectively reviewed to identify all cases of intracranial aneurysms arising from small-caliber vessels (≤ 2 mm) treated with the p48 MW HPC device under SAPT (Prasugrel). The clinical presentation and outcomes, periprocedural and postprocedural complications, and degree of occlusion at follow-up (FU) were evaluated.

**Results:**

A total of 62 patients (70.7% women) with 65 aneurysms were treated. Two patients (3.2%) experienced complications associated with FD use. No cases of aneurysm rupture or hemorrhagic complications associated with antiplatelet therapy or FD treatment were recorded. The rate of complete occlusion was 71.9% in the early FU period (3–6 months) and 86.1% in the initial 12-month period.

**Conclusion:**

p48 MW HPC FDs with prasugrel SAPT showed high safety in the treatment of ruptured and unruptured aneurysms arising from small-caliber vessels (≤ 2 mm), and high occlusion rates at early- and mid-term FU.

## Purpose

Advances in neurointerventional technology in the past three decades have increasingly enabled the treatment of complicated intracranial aneurysms. The introduction of flow diverter (FD) stent implantation has led to substantial developments in these treatments, and this method has recently become one of the most frequently used treatments (1).

Most prior studies have described FD implantation in the internal carotid artery (ICA) or vessels with wide diameter (2, 3). However, the use of FDs in distal localization and vessels with small caliber remains controversial and has not yet been sufficiently evaluated (4–6). In recent years, major advancements have been made in the field of FD stents, and more implants are becoming available for the treatment of aneurysms in small-caliber vessels. Prominent examples include p48 MW HPC (WallabyPhenox, Bochum, Germany), Silk Vista Baby (Balt Group), and FRED Jr. (Terumo Neuro). Recently developed coatings have enabled additional safety in FD treatment of small-caliber vessels. The hydrophilic polymer coating (HPC, WallabyPhenox, Bochum, Germany), a glycan-based multilayer polymer with notable antithrombogenic properties, to date has demonstrated safety and efficacy in FD treatments of ruptured and unruptured aneurysms under single platelet therapy (SAPT) (7).

However, no prior study has specifically analyzed the results of FD treatments in small-caliber vessels (≤ 2 mm) under SAPT. Therefore, the aim of this study was to present our experience in using p48 MW HPC devices under prasugrel (Efient, Daiichi Sankyo, Munich, Germany) SAPT for aneurysms in small-caliber vessels.

## Methods

### Patient selection

We retrospectively reviewed our prospectively maintained database to identify patients with ruptured and unruptured aneurysms in small-caliber vessels who were treated with at least one p48 MW HPC FD with prasugrel SAPT between February 2019 and June 2024.

The present study included only patients with aneurysms in vessels with a diameter ≤ 2 mm, receiving an FD as either an initial treatment or a follow-up (FU) treatment after reperfusion. The deployment of other devices such as coils or similar devices during the procedure was not an exclusion criterion.

The demographic details, aneurysm morphology and location, FD type and size, platelet function test results, intraprocedural and postprocedural complications, clinical outcomes, and radiological FU data were recorded.

### Device description

The p48 MW HPC is composed of 48 braided drawn solid tubular wires, with each strand consisting of platinum-filled nitinol tubing coated with a glycan-based multilayer HPC. The device is compatible with 0.021-inch inner diameter microcatheters, and is available with a nominal diameter of 2 mm or 3 mm, designed to treat vessels with diameters of 1.75 or 3 mm, respectively.

### Endovascular treatment

All treatments were performed in patients under general anesthesia. Arterial access was obtained via a 6–8\u00B0F short sheath (Terumo, Tokyo, Japan), typically in the right groin, and a standard 6–8\u00B0F guiding catheter. In most cases, a 5\u00B0F intermediate catheter (e.g., Navien A Mourant 058, Medtronic, Dublin, Ireland) was used. The FDs were inserted via a 0.021-inch microcatheter of the following types: Headway 21 (MicroVention Terumo, Tokyo, Japan), Prowler Select Plus and Rapid Transit (Cerenovus, Johnson & Johnson, New Brunswick, NJ, United States), Trevo Pro 18 (Stryker Neurovascular, Kalamazoo, MI, United States), or Rebar 18 (Ev3). Heparinized irrigation solutions were used in all catheters, with a dose of 5,000 IU unfractionated heparin per liter. All patients with unruptured aneurysms received intravenous administration of 3,000–5,000 IU heparin. FD diameter and length were selected according to two- or three-dimensional measurements of parent artery diameter.

### Medication

Patients with unruptured aneurysms received either a loading dose of 30–60 mg prasugrel orally (PO) 24–48 h (h) or 1 × 10 mg prasugrel PO daily for a minimum of 5 days before the procedure. The sufficiency of platelet inhibition on the intervention day was assessed with a multiplate analyzer (Roche Diagnostics, Mannheim, Germany) and VerifyNow (Accriva, San Diego, CA, United States). If the prasugrel-SAPT responses were either inadequate or excessive, the doses were modified to twice or half the standard dose, respectively. Postprocedural patients received 10 mg prasugrel PO daily for 6 months; the medication was changed thereafter to 1 × 100 mg acetylsalicylic acid (ASA) PO daily with an overlap of three days. In patients with ruptured aneurysms, a loading dose of 30–60 mg prasugrel PO was administered via a gastric tube 3 h before implantation, or a body-weight-adjusted bolus of the platelet inhibitors eptifibatide (Integrilin; GlaxoSmithKline, Munich, Germany), cangrelor (Kengrexal; iesi Farmaceutici, Parma, Italy), or tirofiban (Aggrastat; Carrevio, Vancouver, Canada) was administered intravenously during the procedure before FD implantation, and a loading dose of 30–60 mg was administered after implantation, with an overlapping, body-weight-matched continuous intravenous infusion of eptifibatide or cangrelor. Postprocedurally, patients received 2 × 20 mg prasugrel PO daily, and platelet inhibition was evaluated with a multiplate analyzer or VerifyNow daily in the first week. The prasugrel dose was optimized or maintained, as indicated by the test results.

### Follow-up

Planned angiographic examinations were conducted at the following time points: early (3–6 months), mid-term (9–18 months), and long-term FU (>19 months). Assessment of aneurysmal occlusion was recorded according to the O’Kelly-Marotta (OKM) scale, on the basis of the degree of aneurysm perfusion (8). The definition of adequate occlusion was set at OKM grades C or D.

Patients with unruptured aneurysms underwent magnetic resonance imaging (MRI) with diffusion-weighted imaging (DWI) and T2/fluid-attenuated inversion recovery within 72 h of the procedure. The DWI lesions were classified as silent emboli, and the territorial T2 lesions were classified as ischemic infarcts. The number of silent emboli and the extent of the infarcts were documented.

Neurological examinations were conducted by a neurologist or a certified stroke nurse within 24 h after the procedure (periprocedural period), as well as during early and long-term FU (postprocedural period). The modified Rankin Scale (mRS) (9) was used to assess outcomes.

### Statistical analysis

Categorical variables were presented as percentages. Continuous variables were presented as means and standard deviations. The Chi-Square and Student’s t-test -tests were used to analyze categorical and continuous variables, respectively. All statistical tests were two-sided, and *p* < 0.05 was considered significant. “IBM SPSS Statistics 26” was the used software for the statistical analysis.

## Results

### Patients and aneurysms

Between February 2019 and June 2024, 62 patients received 65 treatments for aneurysms in small-caliber vessels (≤ 2 mm) with one or more p48 MW HPC FD stents. The mean patient age was 59.8 years. Saccular aneurysms had a mean maximum diameter of 3.1 mm. Demographic, aneurysm, and FD characteristics are summarized in Table 1.

**Table 1:** Demographic data, aneurysms, and FD characteristics.

Patients	
Number of patients/Aneurysms	60/65
Female/Male	41 /19
Age	Mean 59,8 years (range 39–80 years)
Aneurysm/FD	
AcomA	32/65
ACA_A1	3/65
ACA_pericallosal	15/65
ACA, callosomarginal	1/65
MCA_M1	3/65
MCA_M2	2/65
MCA_MCA bif	5/65
BA	1/65
PCA	2/65
PICA	1/65
Aneurysm type	
Blister	7/65
Saccular	58/65
Aneurysm maximum diameter	Mean 3.1 mm (range 0,4–8 mm)
P48 HPC diameter 2 mm	52/65
P48 HPC diameter 3 mm	8/65
P48 HPC diameter 2 and 3 mm	5/65
FD length	Mean 13,9 mm (range 9–18 mm)
Number of FDs per session	Mean 1,1 mm (range 1–2)
EVT	
Simultaneous treatment (coiling)	3/65
Pretreated (coiling)	11/65
Pretreated (FD)	1/65
Pretreated (clipping)	1/65

FD, flow diverter; AcomA, anterior communicating artery; ACA, anterior cerebral artery; MCA, middle cerebral artery; bif, bifurcation; BA, basilar artery; PICA, posterior inferior cerebellar artery; PCA, posterior cerebral artery; HPC, hydrophilic polymer coating.

Of the 65 aneurysms, 46 (70.8%) were unruptured, and 19 (29.2%) were ruptured. In 13 cases (20%), FD implantation was performed as a retreatment for recurrent or residual perfusion (11 after coiling, 1 after FD, and 1 after surgical clipping). In two (3.1%) cases, EVT was performed with FD-assisted coiling. However, the aneurysms were not tightly coiled (Modified Raymond-Roy classification IIIb).

### Technical difficulties

In three cases, implantation of a second FD in telescoping technique was required to achieve sufficient coverage of the aneurysm neck. The initial FD shortened during the implantation process, thus resulting in inadequate coverage of the aneurysm neck. In one of these three cases, the first FD was not fully adapted to the vessel wall after implantation and showed a “fish-mouth” configuration at the distal end of the stent. Therefore, a stent retriever (Solitaire-AB 3/20, Ev3) was inserted for several minutes to enhance wall adaptation. The FD stent then showed adequate wall positioning; subsequently, the second FD was implanted in telescoping technique, and the aneurysm was adequately covered. Despite several challenges, all planned treatments were technically successful. Furthermore, all aneurysms were adequately covered with one or two FDs, and all FDs exhibited sufficient adaptation to the parent vessel.

One patient developed an ICA dissection caused by the guiding catheter. This dissection was not hemodynamically relevant and was covered with two FDs. This complication was not associated with the FD implantation, and did not cause ischemia or neurological deficits in the patient.

### Angiographic follow-up

All patients except one presented at the early FU (98.4%). At the mid-term FU, 32 (49.2%) of the 65 treatments were also angiographically controlled. Notably, all patients underwent angiographic monitoring at least once within the initial year after EVT.

Early FU (FU1, 3–6 months) occurred a median of 6 months post-procedure. Complete aneurysm occlusion (OKM D) was observed in 46/64 (71.9%) cases in early FU. Neck remnants (OKM C) were detected in 11 (17.2%) aneurysms, and seven cases (10.9%) exhibited subtotal aneurysmal filling (OKM B).

Mid-term follow-up DSA revealed OKM D in 26 of 32 (81.2%) and OKM C in 3 of 32 (9.4%) cases.

In the initial 12-month period, 56 of 65 aneurysms (86.1%) demonstrated complete aneurysm occlusion (OKM D). However, seven cases (10.8%) exhibited neck remnants (OKM C), whereas two cases (3.1%) exhibited OKM B.

The results persisted in the most recent follow-up examination (mean 17.7 months; median 15 months). The changes in OKM grades at each follow-up visit are summarized in Table 2.

**Table 2:** OKM grades 1–3, and latest possible follow-ups (FUs).

	*n*	OKM D (n; change)	OKM C (n; change)	OKM B (n; change)	OKM A (n; change)
FU1 (3–6 months) *n* = 64/65 (98.5%)	64	71.9% (46)	17.2% (11)	10.9% (7)	0% (0)
FU2 (9–18 months) *n* = 49/65 (75.4%)	49	87.8% (43; +2 from B;+7 from C)	10.2% (5; +3 from B)	2% (1)	0% (0)
FU3 (>19mo), *n* = 30/65 (46.1%)	30	86.7% (26)	10% (3)	3.3% (1)	0% (0)
Latest possible follow-up, *n* = 65 (100%)	65 (100%)	86,1% (56)	10.8% (7)	3.1% (2)	0% (0)

**Figure 1:**
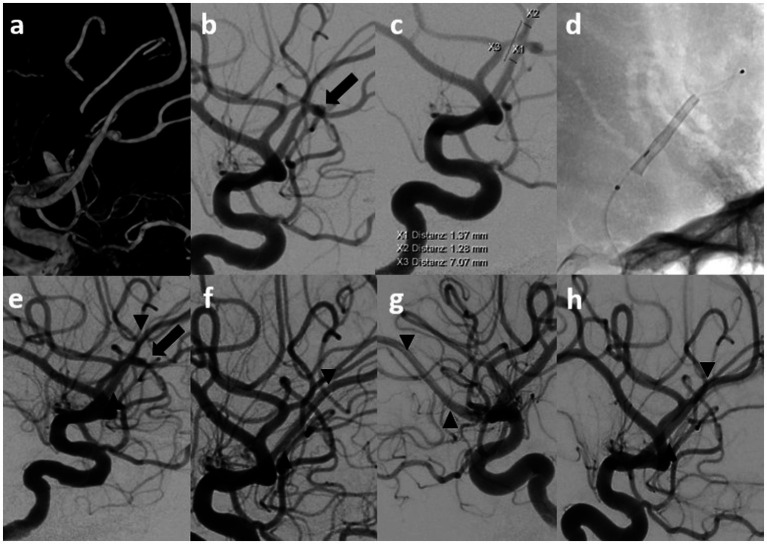
Shows an example of treatments with short- and long-term follow-up. Endovascular treatment (EVT) and diagnostic imaging in a patient with a distal anterior cerebral artery aneurysm. Digital subtraction angiography (DSA) with contrast medium injection into the left internal carotid artery (ICA) in 3D and lateral projections (90°) revealed an aneurysm at the division of the anterior cerebral artery (ACA) into the pericallosal and callosomarginal arteries (arrow), arising from the A2-A3 segment (1.4–1.3 mm) **(a–c)**. Control imaging was performed during and after flow diverter (FD) implantation (P48 MW HPC 2/15 mm) (between the arrowheads) **(d,e)**. The first DSA follow-up after six months revealed complete occlusion of the aneurysm (left ICA; lateral views at 90° and 45°) **(f,g)**. The aneurysm remained completely occluded at the 24-month follow-up **(h)**.

Intimal hyperplasia was observed in four patients in the present study without any evidence of hemodynamic relevance. Notably, all patients were asymptomatic, and intimal hyperplasia demonstrated regressive development over the course of the study. Two cases exhibited complete regression in mid-term follow-up, whereas the remaining two cases demonstrated complete regression in long-term follow-up (Figure 1).

### Peri- and post-procedural complications associated with flow diversion

During the treatment of a non-ruptured AcomA aneurysm with a p48 MW HPC FD, intraprocedural clot formation was detected in a segment of the middle cerebral artery, which was successfully dissolved by intra-arterial administration of 10 mg of eptifibatide. After the treatment, the patient exhibited transient leg paresis, which resolved during the clinical stay. Complication rates, occlusion rates, and DWI lesions according to the location of the unruptured aneurysms are summarized in Table 3.

**Table 3:** Complication rates, occlusion rates and DWI lesions based on location for the unruptured treated aneurysms.

	AcomA	ACA pericallosal	ACA callosomarginal	MCA M1	MCA M2	MCA bif
Number of aneurysms treated	25	12	1	2	2	4
Periprocedural complications	1	0	0	0	0	0
Postprocedural complications	0	0	0	0	0	0
MRI FU	25	11	1	2	2	4
No DWI lesions	7	3	1	2	1	2
1–5 DWI lesions	8	5	0	0	1	2
> 5 DWI	7	3	0	0	0	0
Deterioration of mRS score on discharge	1	0	0	0	0	0
Permanent deterioration of the mRS score	0	0	0	0	0	0
Intimal hyperplasia	1	0	1	0	0	0
OKM D 12 months	21	10	1	2	2	3
Narrowing of covered vessel	0	3	1	0	0	2
Occlusion of covered vessel	0	1	0	0	0	0

AcomA, anterior communicating artery; ACA, anterior cerebral artery; MCA, middle cerebral artery; bif, bifurcation; MRI, magnetic resonance imaging; DWI, diffusion-weighted imaging; mRS, modified rankin scale; OKM, O’Kelly-Marotta.

Postoperative complications (24 h to 30 days) were observed in one patient. This patient exhibited post SAH cerebral vasospasm complication primarily in the anterior cerebral artery (ACA) after the rupture of a pericallosal artery aneurysm, thus resulting in ischemic complications. Complication and occlusion rates according to the locations of the treated ruptured aneurysms are summarized in Table 4.

**Table 4:** Complication and occlusion rates based on location for the treated ruptured aneurysms.

	AcomA	ACA A1	ACA pericallosal	MCA M1	MCA MCA bif	BA	PCA	PICA
Number of aneurysms treated	7	3	3	1	1	1	2	1
Periprocedural complications	0	0	0	0	0	0	0	0
Postprocedural complications	0	0	0	0	0	0	0	0
Deterioration of mRS score on discharge	0	0	0	0	0	0	0	0
Permanent deterioration of the mRS score	0	0	0	0	0	0	0	0
Intimal hyperplasia	1	0	0	0	0	0	0	1
OKM D 12 months	7	3	3	1	1	1	2	1
Narrowing of covered vessel	0	0	1	0	0	0	0	1
Occlusion of covered vessel	0	0	0	0	0	1	0	0

AcomA, anterior communicating artery; ACA, anterior cerebral artery; MCA, middle cerebral artery; bif, bifurcation; BA, basilar artery; PCA, posterior cerebral artery; PICA, posterior inferior cerebellar artery; mRS, modified rankin scale; MRI, magnetic resonance imaging; DWI, diffusion-weighted imaging; OKM, O’Kelly-Marotta.

In total, only two patients (3.1%) showed peri- or post-procedural FD-dependent complications. The analysis revealed no permanent deterioration in mRS scores in any of the patients.

A total of 44 (95.6%) pre-discharge MRI scans after treatment of 46 unruptured aneurysms were available for review. Of these, 38.6% of cases revealed no DWI lesions. In contrast, 44.3% of the MRIs revealed five or fewer DWI lesions, whereas an additional seven (17.1%) revealed more than five DWI lesions. No territorial infarcts, subarachnoid hemorrhages, or intracerebral hemorrhages were detected.

### Statistical analysis

The univariate analysis revealed no statistically significant correlation between complete and non-complete occluded aneurysms in the initial 12 month period. The comparison between completely and incompletely occluded aneurysms in the first 12 months is presented in Table 5.

**Table 5:** Comparison between completely and incompletely occluded aneurysms in the first 12 months.

Variable/Group	Complete occlusion *N* = 54	Incomplete occlusion *N* = 11	*P*
Age (mean, SD)	59.4 (10.5)	61.6 (7.9)	0.501
Male sex (%)	14 (26)	5 (46)	0.194
Morphology			0.206
Saccular (%)	47 (87)	11 (100)	
Blister (%)	7 (13)	0 (0)	
Neck (mean, SD)	2.2 (0.8)	2.3 (0.8)	0.817
Width (mean, SD)	2.7 (1.1)	3.2 (1.9)	0.311
Depth (mean, SD)	2.6 (1.2)	3.2 (1.7)	0.193
Distal vessel diameter (mean, SD)	1.5 (0.3)	1.6 (0.2)	0.283
Proximal vessel diameter (mean, SD)	1.7 (0.3)	1.8 (0.2)	0.469
Number of FDS			02.46
Single (%)	48 (89)	11 (100)	
Telescopic (%)	6 (11)	0 (0)	
Number of FD (mean, SD)	1.1 (0.3)	1.0 (0)	0.253
Length of FD (mean, SD)	13.7 (2.1)	15 (0.0)	0.054
Diameter of FD (2 mm) (%)	48 (89)	8 (73)	0.157
Technical difficulty (%)	3 (6)	0 (0)	0.423
Coverage of side branches (%)	49 (91)	11 (100)	0.294
Additional coiling during the same session	3 (6)	1 (9)	0.657
Previous treatment of the aneurysm	14 (26)	0 (0)	0.057
Ischemic complications (%)	1 (2)	0 (0)	0.649
Other complications (%)	1 (2)	0 (0)	0.649
Lesions on MRI (%)	25 (58)	9 (90)	0.058
Lesions on MRI (mean, SD)	4.5 (12.2)	7.5 (5.3)	0.478
Baseline mRS (mean, SD)	1.5 (1.9)	0.6 (1.5)	0.132
MRS on discharge (mean, SD)	0.7 (1.2)	0.2 (0.4)	0.139

AcomA, anterior communicating artery; ACA, anterior cerebral artery; MCA, middle cerebral artery; bif, bifurcation; MRI, magnetic resonance imaging; DWI, diffusion-weighted imaging; mRS: modified rankin scale; PCA, posterior cerebral artery; PICA, posterior inferior cerebellar artery.

## Discussion

In the past 15 years, numerous studies have reported favorable and safe outcomes of FD treatments for both ruptured and non- ruptured aneurysms, particularly in proximal or large-caliber vessels (3, 7, 10). However, advances in microcatheters and FD developed for small-caliber vessels have enabled the inclusion of FDs as an additional tool in the endovascular repertory. Treatment of small-caliber vessels can pose challenges because of the high risk of injury to these vessels (11). Furthermore, a hemodynamically significant decrease in blood flow is possible if the procedure is prolonged or if iatrogenic vasospasms occur. The small caliber of the vessels and distal location of the aneurysms can also cause difficulties in FD therapy, particularly because of elevated risk of inadequate device deployment and difficulty in performing corrective maneuvers, such as balloon catheter insertion.

However, software and algorithms, specifically designed to improve the accuracy of the device selection procedure, have enhanced the performance of FD treatment and enabled easier, safer device implantation, even in small-caliber vessels (12, 13). In addition, innovative corrective maneuvers are being developed for inadequately inserted FDs with simple-to-insert stents, such as the Stent Retriever or Comaneci device (14, 15).

A limited number of studies have documented the use of FD treatment for distal intracranial aneurysms involving the pericallosal artery, and have reported favorable outcomes with minimal complications (11, 16). However, no substantial experience in the treatment of aneurysms in distal segments or small-caliber vessels under SAPT has been reported. HPC is a hydrophilic glycan-based multilayer polymer coating that can be applied to nitinol surfaces. This low-thrombogenic coating is currently the only type indicated for use with SAPT.

Prasugrel SAPT in the treatment of ruptured and non-ruptured aneurysms with HPC-coated FDs has been shown to be safe and effective (7, 17). In contrast, Aguilar et al. have reported elevated rates of thromboembolic and ischemic complications with ASA (18). Therefore, prasugrel is also the drug of choice for the treatment of aneurysms in small-caliber vessels. Prasugrel has also been shown to be safe in surgical procedures with SAPT (19).

In this cohort, the complete aneurysm occlusion rate (OKM D) was 72.3% in the first 3 months, and a further 16.9% showed only neck remnants (OKM C). In the first 12 months, the occlusion rate increased to 86.1%, and a further 10.8% showed an OKM C score. Consequently, the p48 HPC FD under SAPT exhibited adequate protection (OKM C + D) of 89.2% in the initial 3–6 months and 96.9% in the first 12 months. The occlusion rates in this cohort exceeded those attained with uncoated p48 FD treatments. In contrast, Al Matter et al. have reported complete and adequate occlusion rates of 47.9 and 64.6%, respectively, in the first 12 months (20). However, importantly, this study included aneurysms in vessels with diameters greater than 2 mm.

In current study, a complete occlusion rate of 86.1% was observed in DSA in the 12 months after FU implantation. These results are consistent with those of previous studies. For instance, Hanel et al. (10) and Taschner et al. (21) have documented complete aneurysm occlusion rates of 82 and 81.9%, respectively, after implantation of the Pipeline device (Medtronic, Dublin, Ireland) and the Derivo embolization device (DED, Acandis, Pforzheim, Germany). Aguilar et al. (3) have documented similar outcomes, with a complete occlusion rate of 76.6% in 617 aneurysms treated with uncoated p64 devices in mid-term FU. Pierot et al. (22) have also reported an occlusion rate of 73.3% in 103 patients with intracranial aneurysms at 12 months. The findings of this study are consistent with those of studies focusing on treatments in both proximal vessels and distal vessels. For example, in the series reported by Dabus et al. (16), the closure rate was approximately 75% in the treatment of 20 complex ACA aneurysms, including six pericallosal A2 aneurysms, and in a study by Khanafer et al., the complete occlusion rate at mid-term FU was 78% in the treatment of 41 distal anterior cerebral artery aneurysms with FD (3).

We observed only two cases (3.1%) with ischemic and thromboembolic complications. The first patient was treated with a p48 HPC FD after aneurysm rupture at the bifurcation of the pericallosal artery and the collosomarginal artery. The FD covered the callosomarginal artery. The patient had severe vasospasm of the ACA. MRI performed 2 weeks after the treatment to clarify the progressive headache and nausea over 2 days showed cerebral ischemia in the area supplied by the callosomarginal artery. The second patient was treated for an AcomA aneurysm by implantation of a p48 HPC in the anterior cerebral artery. During the treatment course, thrombus formation in the middle cerebral artery was observed at the end of treatment control. The patient was treated with an intergrilin IV bolus. Postprocedurally, patient showed mild leg paresis and neglect. The symptoms resolved, and the patient was discharged with mRS 1 and was completely asymptomatic at the 6-month follow-up.

No rerupture, intra- pr extracranial hemorrhage complications were documented in patients before or after discharge.

In the present study, 95% of patients undergoing treatment for a non-ruptured aneurysm underwent an MRI scan in the first 72 h after therapy. Of these, 61.4% exhibited silent DWI lesions, although more than five lesions were detectable in only seven cases (17.1%). In prior studies silent ischemic events were reported in 50.9–90% of cases involving the implantation of the uncoated Pipeline FD (23–25). In other studies using the coated Pipeline FD (Shield coated with phosphorylcholine polymer), fewer DWI lesions (18.2%) were documented, but the treatments were performed under DAPT (26).

### Limitations

This single-center, retrospective, single-arm design study was subject to several inherent limitations. The results are applicable only to the p48 MW HPC FD under Prasugrel as SAPT and cannot be generalized to other FDs, FD-associated procedures, or medications. The assessment of clinical outcomes in patients with ruptured aneurysms is complicated, and the true effects of device-associated complications on the final clinical outcomes are difficult to determine.

## Conclusion

In this study, the use of p48 MW HPC FDs with prasugrel SAPT for intracranial aneurysms arising from small-caliber vessels (≤ 2 mm) was associated with safety, in terms of hemorrhagic complications and very low rates of ischemic complications. Use of p48 MW HPC FDs with prasugrel SAPT achieved a high rate of complete angiographic occlusion at early- and mid-term follow-up.

## Data Availability

The datasets presented in this article are not readily available because of patient data protection. Requests to access these datasets should be directed to Ali Khanafer, mr-khanafer@hotmail.com.
